# Actinomycetes of secondary metabolite producers from mangrove sediments, Central Java, Indonesia

**DOI:** 10.14202/vetworld.2021.2620-2624

**Published:** 2021-10-07

**Authors:** Wilis Ari Setyati, Delianis Pringgenies, Nirwani Soenardjo, Rini Pramesti

**Affiliations:** Department of Marine Science, Faculty of Fisheries and Marine Science, Diponegoro University, Semarang, Central Java, 50275, Indonesia.

**Keywords:** actinomycetes, biosynthetic gene cluster, Karimunjawa Island, *NRPS*, *PKS*

## Abstract

**Background and Aim:**

Actinomycetes are a group of Gram-positive bacteria with a fungus-like morphology. Their natural habitat encompasses terrestrial and water areas, including mangrove ecosystems. This study aimed to assess the *PKS* and *NRPS* genes as the producers of secondary metabolites and to determine the target bacterial species using molecular DNA tests.

**Materials and Methods:**

In this study, we isolated bacteria from sediment samples from mangrove forests located on Karimunjawa Islands and in Semarang city, purified bacteria, screened for antibacterial activity, extracted bacterial DNA, amplified the *NRPS* gene, detected and amplified the *PKS-I* and *PKS-II* genes, amplified and sequenced the 16S rRNA, processed molecular data, and simulated a map of secondary metabolite producing genes.

**Results:**

Samples from the Karimunjawa Islands yielded 19 bacterial isolates, whereas samples from Semarang yielded 11 bacterial isolates after culture in different media. Further experiments identified three active isolates, which were termed PN.SB.6.2, S.SK.6.3, and S.SK.7.1, against pathogenic species of *Escherichia coli*, *Staphylococcus aureus*, and *Listeria monocytogenes*. Isolate PN.SB.6.2 was determined to possess three biosynthetic gene clusters (BGCs), whereas the remaining two isolates, S.SK.6.3 and S.SK.7.1, only possessed two BGCs, namely, *NRPS* and *PKS II*.

**Conclusion:**

Products were estimated to be in the *NRPS*, thiopeptide, RiPP-like, siderophore, betalactone, terpene, Type III *PKS*, CDPS, and lassopeptide groups. DNA identification of the isolates found three species of actinomycetes with antibacterial potential, namely, *Virgibacillus salaries*, *Bacillus licheniformis*, and *Priestia flexa*.

## Introduction

Pathogenic bacteria are microbes that are capable of causing disease and can spread through the human population in a variety of ways. The solution for infectious diseases caused by pathogenic bacteria is the use of antibiotics, which are drugs that have been specially formulated to kill bacteria. Antibiotics are a group of chemical compounds that are produced organically or manufactured synthetically and can inhibit the growth of or eradicate bacteria or other organisms. Actinomycetes are a group of useful bacteria with a widespread distribution that is commonly found in terrestrial habitats [1,2]. They are characterized as Gram-positive, filamentous, spore-forming bacteria and have G+C content in their DNA (57-75%). Moreover, in the past, actinomycetes were designated as an intermediate species group between bacteria and fungi, but are currently classified as prokaryotes. Most species of actinomycetes live independently as saprophytic bacteria in soil and water, and they often associate with vascular plants [3,4]. Actinomycetes are members of the order Actinomycetales. This order consists of three families, namely: (1) *Mycobacteriaceae*: The cells of these microorganisms do not produce a mycelium or produce a rudimentary mycelium. Bacteria from *Mycobacterium* and *Mycococcus* genera fall into this category [5]; (2) *Actinomycetaceae*: The members of this family produce spores and are motile. The *Actinomyces* and *Nocardia* genera are among the members of this family [6]; and (3) *Streptomycetaceae*: The members of this family form an undivided vegetative mycelium. The *Streptomyces*, *Micromonosora*, and *Thermoactinomyces* genera are members of this family [7].

Actinomycetes synthesize useful antimicrobial compounds. Actinomycetes samples from the soil of various vascular plants showed antibiotic properties against the bacteria *Escherichia coli* and *Staphylococcus aureus* and the fungi *Trichophyton mentagrophytes* and *Candida albicans* [8,9]. Therefore, mangrove areas are thought to have potential as a habitat for actinomycetes. Mangrove forests are known habitat for organisms and a source of nutrition, elements, and amino acids [10-13]; moreover, they provide bacterial diversity [14]. The research and discovery of new types of antibiotics are extremely time-consuming and costly [15]. In an effort to discover alternative sources of antibiotics, the rediscovery of known compounds is a phenomenon that scientists encounter often. The rapid development of the genomics, bioinformatics, metabolic engineering, and synthetic biology fields provides new opportunities for the discovery of new compounds for use as antibiotics and other pharmaceutical products [16].

This study aims to detect the *PKS* and *NRPS* genes as producers of secondary metabolites from culturable actinomycetes and to determine the target bacterial species using molecular DNA tests.

## Materials and Methods

### Ethical approval

The study was approved by the Ethics Committee of the Faculty of Fisheries and Marine Sciences, Diponegoro University, Indonesia (4099/UN7.5.10.2/PP/2021).

### Study period and location

The samples were collected from September to December 2020 in the forests of Nyamuk Island, Karimunjawa Islands, Jepara Regency, and Tapak village, Tugurejo sub-district, Semarang city. For the sake of brevity, the group samples from Nyamuk Island will be referred to as the “Karimunjawa samples” and those from the Tapak village will be referred to as “Semarang samples” hereinafter.

### Bacterial isolation and mangrove sediment samples

Treatment of sediment samples up to bacterial isolation was carried out according to the method described in Davies-Bolorunduro *et al*. [17], with several modifications. Bacteria were isolated using the spread plate method with stratified dilution. From each dilution, 50 μL was taken and was spread on the surface of five different types of media. The media used were marine agar (Zobell), International Streptomyces Project 1 (ISP 1), humic acid vitamin agar (HVA) [18], and two modified media, that is, the ISP 1 1 + humic acid (HA) and marine agar + HA (Zobell + HA). Nystatin (60 mg/L) was introduced into each medium, and all media were incubated at 29-34°C for 1-7 days.

### Purification of bacteria and screening of antibacterial activity

The bacterial isolates were purified according to Davies-Bolorunduro *et al*. [17]. The Actinomycetes that developed on the isolation media were grouped according to their morphology. Each isolate was grown on new media using the streak plate method followed by reincubation at 29-34°C for 1-5 weeks. The bacterial screening described here refers to the Kirby–Bauer method, with several modifications [19]. Two-week-old bacteria cultured on the disks were then placed onto the surface of Mueller-Hinton agar plates impregnated with the test pathogenic bacteria (*S. aureus*, *E. coli*, *Listeria* *monocytogenes*, and *Enterobacter aerogenes*).

### Bacterial DNA extraction

The Chelex method was employed for DNA extraction [20]. Fifty microliters of Aqua Bidest and 1 mL of 0.5% saponin were added to PBS. This mixture was incubated overnight at 4°C. After incubation, the mixture was homogenized using a centrifuge at a speed of 12,000 rpm for 10 min. The resulting supernatant was then removed and 1 mL of PBS was added to the mixture before reintroduction into the centrifuge. The resulting supernatant was discarded to remove saponin. Subsequently, another 100 μL of Aqua Bidest and 50 μL of Chelex 20% were added to the mixture, which was then heated for 10 min and placed in a vortex machine for 5 min. The preparation was then processed in a centrifuge at 12,000 rpm for 10 min. The extract was kept in a freezer for 24 h for the determination of DNA quality using a nanodrop device.

### *NRPS* gene amplification and *PKS-I* and *PKS-II* gene detection and amplification

*NPRS* gene detection was performed using a PCR method with a primer pair consisting of A2gam F (5’-AAGGCNGGCGSBGCSTAYSTGCC-3’) and A3gamR (5’-TTGGGBIK BCCGGTSGINCCSG AGGTG-3’) [21]. *PKS-I* and *PKS-II* gene detection and amplification were carried out according to the method described in El Samak *et al*. [22].

### 16S rRNA amplification and sequencing

The DNA amplification performed in this study was a replication of the method reported in Radjasa *et al*. [23]. The primers used were as follows: Forward: 5’-AGAGTTTGATCMTGGCTCAG-3’, positions 8-27 and 1500; reverse: 5’-GGTTACCTTGTTACGACTT-3’, positions 1510–1492 based on the sequence of the 16S rRNA from *E. coli* [24]. The PCR DNA amplification method was carried out on a thermal cycler (Mini cycler TM, MJ Research Inc., Watertown, MA, USA) with the following temperature conditions: Initial denaturation at 94°C for 2 min; followed by 30 cycles of denaturation at 94°C for 2 min, annealing at 45°C for 2 min, and extension at 72°C for 2 min; and a final extension at 72°C or 3 min. Electrophoresis was carried out by inserting 1 μL of the PCR product aliquot into a well that was filled with 1% agarose gel. The preparations were then placed in 50× TAE buffer and observed to determine whether the DNA amplification was satisfactory. The products of PCR amplification were purified and concentrated using a Microcon-100 microconcentrator (Amicon, Beverly, MA, USA) according to the OEM manual. Finally, *16S rDNA* gene sequences were determined using the SequiTherm Long-Read Sequencing Kit (Epicenter Technologies, Madison, WI, USA).

### Molecular data processing and genetic mapping simulation of secondary metabolite producers

The data obtained from sequencing were edited using the MEGA 7.0 software. The data from the *16s rDNA* sequencing were then matched with data from the NCBI GenBank. Whole-genome sequencing data from the same species as that of the candidate obtained based on molecular identification were downloaded from GenBank. The data were then submitted to AntiSMASH, to obtain an approximate map of the genes that produce secondary metabolites.

## Results

### Bacterial isolation and antibacterial activity

**Figure-1:**
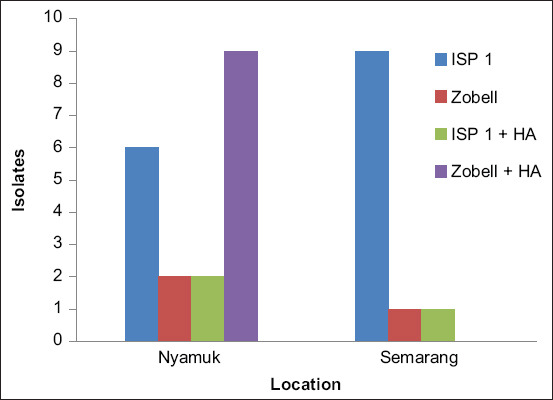
The number of isolates successfully obtained from Karimunjawa and Semarang based on culture media.

The isolation of actinomycetes from samples resulted in 19 isolates from the Karimunjawa Islands and 11 isolates from Semarang. Bacterial isolation from the mangrove sediments collected in Semarang and the Karimunjawa Islands was performed using four types of media. Together, these media yielded 30 isolates. The ISP 1 medium yielded in nine Semarang and six Karimunjawa isolates. The ISP1 + HA gave one Semarang isolate and two Karimunjawa isolates, whereas the Zobell + HA only yielded nine Karimunjawa isolates. The Zobell medium gave the greatest number of isolates; however, all of them exhibited low biodiversity. In contrast, only specific bacteria from the soil were able to grow on selective media (media containing HA). This is because HA is an important component of soil, as presented in Figure-1.

Screening for antibacterial activity against several pathogenic species, that is, *S*. *aureus*, *E. coli*, and *L. monocytogenes*, identified three isolates that actively produced antibiotic compounds, as presented in Table-1. Three bacterial isolates from mangrove sediment samples exhibited antibacterial properties. The Karimunjawa isolate that was able to inhibit the growth of pathogenic bacteria was isolate number PN.SB.6.2, with activity against *L. monocytogenes*. The Semarang isolate that afforded antibiotic susceptibility toward the pathogenic species included in this study was isolate S.SK.6.3, with activity against *E. coli* (Gram-negative bacterium), *S. aureus* (Gram-positive bacterium), and *L. monocytogenes* (Gram-positive bacterium). Another Semarang isolate, S.SK.7.1, exhibited antibacterial activity against *S. aureus*. The results of this screening also revealed that S.SK.6.3 is a bacterial isolate with wide spectrum antibacterial properties.

**Table-1:** Antibacterial activity screening of Karimunjawa and Semarang isolates.

Isolate code	Origin	Inhibition zone against pathogenic bacteria (cm)		

		*Escherichia coli*	*Staphylococcus aureus*	*Listeria* *monocytogenes*
PN.SB.6.2	Karimunjawa	-	-	1.69±0.36
S.SK.6.3	Semarang	0.84±0	0.60±0.08	1.17±0.58
S.SK.7.1	Semarang	-	0.90±0.07	-

### Biosynthetic gene cluster (BCG) detection and 16S rRNA-based molecular identification of active isolates

BCGs screening, including the *NRPS*, type I *PKS*, and type II *PKS* genes, of the Karimunjawa Islands and Semarang isolates resulted in the data that are presented in Table-2. Isolate PN.SB.6.2 was determined to possess three BGCs, whereas the remaining two isolates, S.SK.6.3 and S.SK.7.1, only possessed the *NRPS and PKS II* genes.

**Table-2:** BGC screening.

Isolate code	Coding genes for secondary metabolites		

	*NRPS* gene	*PKS I* gene	*PKS II* gene
PN.SB.6.2	√	√	√
S.SK.6.3	√	√	√
S.SK.7.1	√	√	√

BGC=Biosynthetic gene cluster

The *16S rRNA*-based molecular identification data from the Karimunjawa and Semarang isolates are presented in Table-2. The active bacterial isolate coded as PN.SB.6.2 was identified as *Virgibacillus salarius*, with 100% matching to the data access number LC537902.1. In turn, the bacterial isolate coded as S.SK.6.3 was identified as *Priestia flexa*, with 100% compatibility to the data access number MT279468.1. Finally, the bacterial isolate coded as S.SK.7.1 was identified as *Bacillus licheniformis*, with 100% compatibility to the data access number MT642946.1 (Table-3).

**Table-3:** Molecular identification of Semarang and Karimunjawa isolates.

Isolate code	Species identification (BLAST)	Accession no.	Sequence length (bp)	Identity (%)	Query cover (%)
PN.SB.6.2	*Virgibacillus salarius*	MW990225	480	100%	100%
S.SK.6.3	*Priestia flexa*	MW990226	1402	100%	100%
S.SK.7.1	*Bacillus licheniformis*	MW990227	787	100%	100%

### BGC mapping simulation using AntiSMASH 6.0

Mapping simulation of BGCs using AntiSMASH 6.0 in the whole genome of the species that matched *P. flexa* (S.SK.6.3) provided six regions producing secondary metabolites (Table-4). Estimates of the resulting products include lassopeptide, terpene, siderophore, *NRPS*, and Type III *PKS* groups.

**Table-4:** *Bacillus flexus*/*Priestia flexa* (NZ_FMBD01000005.1).

Region	Region location (nucleotides)	Type	Most similar known cluster	Similarity
Region 5.1	12,591-36,521	Lassopeptide	Paeninodin	80%
Region 5.2	41,198-63,054	Terpene	-	-
Region 6.1	37,947-53,928	Siderophore	-	-
Region 11.1	1-39,588	NRPS	Bacillibactin	53%
Region 24.1	1-28,758	Type III PKS	-	-
Region 52.1	1-23,842	NRPS	-	-

## Discussion

*V. salarius* is a member of the *Virgibacillus* genus and is a Gram-positive bacterium [25]. The species is known to produce compounds with antibacterial properties [26,27]. *P. flexa*, previously known as *Bacillus flexus*, is a Gram-positive bacterium from the genus *Priestia*. The previous studies reported that this species also shows antibacterial properties against other pathogenic species [28]. *B. licheniformis* is a species that is found abundantly in soil. This bacterium is prized in the industrial sector for its ability to produce the alkaline enzyme serine protease [29]. In addition, the species has also been reported to possess antibiotic activity with a large spectrum [30]. The data obtained from the 16S rRNA sequencing of the three samples have been deposited in GenBank, with access numbers MW990225, MW990226, and MW990227.

Region 11.1 had 53% match with the gene clusters of organisms that produce Fe ion-binding compound (bacillibactin) [31]. Several other regions have not been determined to have any matches with specific secondary metabolite producing genes. Mapping simulation of BGCs in the whole genome of the same species with *B. licheniformis* (S.SK.7.1) samples identified 10 regions of secondary metabolite producing gene clusters. The resulting products were estimated to be in the *NRPS*, thiopeptide, RiPP-like, siderophore, betalactone, terpene, Type III *PKS*, CDPS, and lassopeptide groups. Two gene clusters were determined to have similarities with the antibiotic-producing gene clusters.

Among all of these secondary metabolite-producing regions, gene clusters were identified that were similar to the antibiotic-producing gene clusters, Region 5.1, which exhibited an 80% match with the paeninodin producing gene clusters [32], as shown in Table-4. Region 2 exhibited a 7% match with gene clusters producing butirosin A/B [33] and lichenicidin VK21 A1 [34], as shown in Table-4.

## Conclusion

Thirty isolates of actinomycetes were identified in this study; 19 of them were from the Karimunjawa sample and 11 of them were from the Semarang sample, after culture on different media. Among the isolates identified here, three showed antibacterial activity against the *E. coli* (Gram-negative bacterium), *S. aureus* (Gram-positive bacterium), and *L. monocytogenes* (Gram-positive bacterium) pathogenic species. Isolate PN.SB.6.2 possessed three BGCs, whereas the remaining two isolates, S.SK.6.3 and S.SK.7.1, only possessed two BGCs, namely, *NRPS* and *PKS* II. DNA identification of the isolates yielded three species of actinomycetes with antibacterial potential, namely, *Virgibacillus salaries*, *B. licheniformis*, and *P. flexa*.

## Authors’ Contributions

WAS and DP: Designed the study. NS: Prepared the samples. WAS, DP, and RP: Performed data collection, statistical analysis, data interpretation, and manuscript writing. WAS and DP: Supervised the study and manuscript editing. All authors read and approved the final manuscript.
